# Cloning, Expression Analysis and SNP Screening of the *kiss1* Gene in Male *Schizothorax biddulphi*

**DOI:** 10.3390/genes14040862

**Published:** 2023-04-03

**Authors:** Zhulan Nie, Nianhua Zhao, He Zhao, Zhengyi Fu, Zhenhua Ma, Jie Wei

**Affiliations:** 1 College of Life Sciences and Technology, Tarim University, Alaer 843300, China; 2 Key Laboratory of Tarim Animal Husbandry Science and Technology, Xinjiang Production & Construction, Alaer 843300, China; 3 State Kay Laboratory Breeding Base for the Protection and Utilization of Biological Resources in Tarim Basin Co-Funded by Xinjiang Corps and the Ministry of Science and Technology, Tarim University, Alaer 843300, China; 4 Tropical Aquaculture Research and Development Center, South China Sea Fisheries Research Institute, Chinese Academy of Fishery Sciences, Sanya 572018, China; 5 College of Science and Engineering, Flinders University, Adelaide, SA 5001, Australia

**Keywords:** association analysis, gene expression, kisspeptin, polymorphism, *Schizothorax biddulphi*

## Abstract

*Schizothorax biddulphi* is an endemic fish distributed only in southern Xinjiang, China. Due to overfishing, water conservancy facilities, and other factors, as well as inherent biological limitations, resource recovery is quite difficult. For endangered fish with slow growth, late sexual maturity, and insufficient natural population supplementation, large-scale artificial reproduction and breeding are important for restoring resources. Therefore, it is urgent to optimize the reproductive regulation methods of the fish. The kiss1 gene is a key regulator of the reproductive regulation cascade, and identifying and analyzing the role of kiss1 are important for further elucidating the reproductive mechanism of *S. biddulphi*. To understand the characteristics of the kiss1 of *S. biddulphi*, the full-length cDNA sequence of kiss1 was obtained in this study, and its tissue expression specificity and association with phenotypic traits were analyzed in male fish. The full-length cDNA sequence of kiss1 in *S. biddulphi* was 658 bp, with an ORF of 327 bp, and encoded a 108-amino acid, unstable protein. Homology results indicated that kiss1 was highly conserved. qPCR showed kiss1 expression in different tissues in male *S. biddulphi*, with the highest expression in the gonads, followed by muscle, and significantly lower expression in the swim bladder, pituitary gland, heart, hypothalamus, gill, fin, liver, eye, and mid-kidney. qPCR revealed three SNP loci in the exonic region of kiss1. The c.3G>T locus was significantly correlated (*p* < 0.05) with gonad mass and the maturation coefficient in *S. biddulphi*. These results will help uncover the reproductive endocrinology network of *S. biddulphi*, improve artificial breeding technology for fish, and unveil new directions for breeding excellent strains of *S. biddulphi* and molecular marker-assisted breeding.

## 1. Introduction

The Tarim schizothoracin (*Schizothorax biddulphi*) in order Cypriniformes, family Cyprinidae, subfamily Schizothoracinae, and genus *Schizothorax* [1], is a special fish in Xinjiang. It is one of the most representative fish in the Tarim River basin and was once among the most important economic fish in Bosten Lake [2]. In the 1970s, *S. biddulphi* experienced a sharp decline in germplasm resources and numbers due to its biological features, such as slow growth, late sexual maturity, low fertility, and strict requirements for the spawning environment and living conditions, as well as constraints imposed by anthropogenic and natural conditions, resulting in severe deterioration of its habitat [3]. Its distribution is now extremely narrow, with individuals inhabiting only the Tarim River system and in very small numbers. In 1998, this species was included in the Red Book of Endangered Animals of China as a Class II protected wild animal in Xinjiang [4]; in 2004, it was listed as a Class II aquatic animal in Xinjiang Uygur Autonomous Region, and in 2021, it was under second-class state protection, becoming one of the rare fishes endemic to China [5]. Therefore, analyzing *S. biddulphi* reproduction-related genes and screening molecular markers associated with reproduction will have a positive effect on the conservation of *S. biddulphi* as well as resource recovery and species breeding.

Kisspeptin is a polypeptide-like protein encoded by the *kiss* gene that is involved in the regulation of the hypothalamic–pituitary–gonadal axis function in animals and plays an important role in the regulation of reproductive metabolism, puberty initiation, photoperiodic reproduction, and sexual differentiation of the brain [6,7]. The successful identification of kisspeptin and its receptor gene, *GPR54*, as an important neural signaling system [8] was a turning point in neuroendocrinology and redirected animal reproduction research. In sexually mature fish, kisspeptin treatment led to increased gonad weight and gonadosomatic index in some white bass (*Morone chrysops*) and striped bass (*Morone saxatilis*) [9]. The Kisspeptin system of teleosts generally consists of *kiss* genes (*kiss1* and *kiss2*) and kiss receptors (*kissr1*, *kissr2,* and *kissr3*) [10,11]. Most bony fishes, such as goldfish (*Carassius auratus*), zebrafish (*Danio rerio*), and medaka (*Oryzias latipes*), contain both the *kiss1* and *kiss2* genes [10,12]. In almost 20 fish species, only *kiss2* can be found [13,14]. To test whether *kiss* and/or *kissr* have been lost in some species requires a syntenic analysis of *kiss* and *kissr* neighboring genes [15]. For example, in turbot (*Scophthalmus maximus*), kiss1/kissr3, which were thought to be lost in flatfish species, were cloned and functionally characterized using this approach [16]. The actions of different kisspeptins and their receptors vary depending on the species, sex, and developmental stage of the fish. For example, in zebrafish, *kiss 1* is involved in the activation of puberty, while *kiss 2* is involved in the regulation of seasonal reproduction [17]. In goldfish, *kiss 1* is involved in the induction of spawning behavior, while *kiss 2* is involved in the regulation of gonadotropin-releasing hormone (GnRH) secretion [18,19]. Furthermore, the actions of fish kisspeptins can also vary depending on their target tissues. For instance, in medaka, *kissr1* is present in both the brain and the gonads, suggesting a role in both the central regulation of reproduction and the direct regulation of gonadal function [20]. In contrast, in *Salmo salar*, *kissr1* is mainly expressed in the brain, suggesting a more central role in the regulation of reproduction [21].

Although both *kiss1* and *kiss2* are associated with reproductive regulation in teleosts, *kiss1* and its receptor have been found to be the main factors regulating gonadal development in many fish. For example, the mRNA expression levels of kiss1 or *kissr1* fluctuated greatly at different gonadal developmental periods in European sea bass (*Dicentrarchus labrax*) and striped bass [22,23]; in chub mackerel (*Scomber japonicus*) and zebrafish, the expression levels of kiss1 mRNA also fluctuated greatly before and after spawning [24,25]. The *kiss1* gene was originally discovered in a study of human melanoma pigment cells. Because of its tumor metastasis inhibitory effect, it was the first in this system to be studied for its role in tumor proliferation and spread. A large number of studies later reported that mutations in the GPR54 gene lead to incomplete reproductive function in humans and mice and knocking out the kiss1 gene has the same consequences [26]. Some scholars have also found that the *kiss1* gene promotes the onset of puberty [14]. Around puberty, the *kiss1* gene increases its expression level in the hypothalamus of animals by promoting the release of gonadotropins, all of which indicate that the *kiss1* gene plays a role in the regulation of reproduction and demonstrate that regular reproductive activity in animals requires the participation of the reproductive system together with the nervous and endocrine systems. To date, kiss genes have been identified in zebrafish, medaka, European sea bass, goldfish, chub mackerel, Japanese puffer (*Takifugu rubripes*), Japanese sturgeon (*Acipenser schrenckii*), pompano (*Trachinotus ovatus*), turbot (*Scophthalmus maximus*), Chinese rare minnow (*Gobiocypris rarus*), and cavefish (*Sinocyclocheilus tingi*) [27,28,29,30,31,32]. The characteristic sequences of the gene and its expression in many tissues have been investigated, but reports on the association between *kiss1* gene polymorphism and reproductive traits are limited.

Single nucleotide polymorphisms (SNPs) are ideal genetic markers with promising applications in genetic diversity analysis, association analysis, variety identification, construction of high-density genetic linkage maps, and assisted breeding [33,34,35,36]. Therefore, in this study, we performed cloning, sequence alignment, and tissue differential expression analysis of the *S. biddulphi kiss1* gene and conducted the first case of molecular marker screening for *kiss1* gene polymorphism and reproductive traits in *S. biddulphi*, thereby providing technical support and a theoretical basis for molecular marker-assisted breeding in *S. biddulphi.*

## 2. Materials and Methods

### 2.1. Test Animals and Sample Collection

*S. biddulphi* were collected from August 2020 to August 2021 in the Kizil River with a geo-cage net (net length 10 m, 33 sections and 20 holes, frame 2 cm × 3 cm, mesh 4 mm). A total of 128 fish were caught, including 75 males and 53 females, with body lengths ranging from 95.05 to 328.52 mm and body masses from 8.02 to 415.02 g. Before the experiment, these fish were placed in a 2 m × 1 m tank for two weeks, and the experiment was carried out using an HZ-060 aerator for oxygenation. Floating compound feed (Sichuan Sterga Feed Ltd., Chengdu, China) was provided three times a day (9:30, 13:30, and 19:30). Fish were fed the diet at 3.0% to 5.0% of their total weight. Due to the small number of females, only a total of 62 males that developed to stage IV or V were selected (developmental stage I-III males were immature, and gonadosomatic indexes could not be calculated). The body lengths of males used in this study were 164.78 ± 32.52 mm, and the total weight was 63.85 ± 44.15 g. Such males were identified based on milky white spermatozoa and a small amount of semen flowing from the abdomen when lightly pressed. Morphological data were collected, and the fish were anesthetized using MS-222 and dissected to take the heart, liver, mid-kidney, muscle, gonads, eye, hypothalamus, pituitary gland, gill, fin, and swim bladder tissues, while the male gonads were weighed to calculate their maturation coefficients. The samples were immediately placed in liquid nitrogen and subsequently stored in a −80 °C freezer. A portion of the gonadal mixture was immersed in centrifuge tubes with anhydrous ethanol and stored in a −20 °C freezer. All animal experiments conformed to the standards of the Committee of Laboratory Animal Experimentation at the China Academy of Agriculture Sciences, China.

### 2.2. Methods

#### 2.2.1. Cloning and Sequence Analysis of the *kiss1* Gene

Total RNA was extracted from the gonadal tissue of *S. biddulphi* using TRIzol reagent (Invitrogen, Waltham, MA, USA) according to the manufacturer’s protocol, and contaminating genomic DNA was removed by treatment with DNaseI (TaKaRa, Shanghai, China). The quality of RNA was examined by spectrophotometry and agarose gel electrophoresis, and the first strand of cDNA was synthesized from the total RNA of mixed gonadal samples according to the manufacturer’s 5′/3′ RACE protocol and stored at −20 °C. Primers were designed using Primer Premier 6.0 based on a comparison of the coding regions of the *kiss1* genes of *C. auratus* (NC_039278.1), *D. rerio* (NC_007122.7), and *O. latipes* (NC_019863.2) in the NCBI database. The primer names and sequences are shown in Table 1.

Specifically, the first round of PCR for 5′ RACE was performed using the primers rkiss1-R1 and rkiss1-R2. The amplification procedure was as follows: 94 °C predenaturation for 2 min, followed by 38 cycles of 94 °C denaturation for 30 s, 70 °C for 30 s, and 66 °C for 30 s. The products of the first round of PCR were diluted 10-fold, and the second round of PCR was performed with conditions of 94 °C predenaturation for 2 min, 94 °C for 30 s, and 66 °C for 30 s. 3′ RACE was performed using the primers rkiss1-F1 and rkiss1-F2 with the same reaction conditions and amplification procedure. PCR products were detected with 1.2% agarose gel electrophoresis and determined to have specific amplification. After specific amplification, cut-gel recovery and T vector splicing was performed, and the ligated products were cloned into the vector and sequenced separately. All sequencing was performed on an ABI377 DNA sequencer (Perkin-Elmer, Norwalk, CT, USA).

#### 2.2.2. Sequence Analysis of the *kiss1* Gene

The intermediate fragment and 5′ RACE and 3′ RACE ends of the obtained *kiss1* gene were spliced using SeqMan to obtain the full-length cDNA of *kiss1*. The open reading frame (ORF) was queried for the *kiss1* gene using DNAStar’s EditSeq program, and the amino acid sequence was translated; the physicochemical properties of the protein and the secondary structure of the protein were analyzed using ExPASy (http://www.expasy.org, accessed on 28 February 2022), NetSurfP 2.0 (https://services.healthtech.dtu.dk/service.php?NetSurfP2.0, accessed on 28 February 2022) and SOPMA (http://metadatabase.org/wiki/SOPMA, accessed on 28 February 2022), and the tertiary structure of the protein was predicted using Phyre2. SignalP5.0 Server (http://www.cbs.dtu.dk/services/SignalP/, accessed on 28 February 2022) was used for signal peptide analysis, NetPhos3.1 (http://www.cbs.dtu.dk/s.ervices/NetPhos, accessed on 28 February 2022)for protein phosphorylation site prediction, TMHMM for transmembrane structural domain prediction, the Cell-PLoc 2.0 package for *kiss1* subcellular localization, NCBI Blast for homology matching analysis, DNAMAN (version v 10.0.2.128; Lynnon Biosoft, Vaudreuil, QC, Canada) for multiple amino acid sequence comparison, and MEGA 7.0 (https://www.megasoftware.net/, accessed on 28 February 2022) for phylogenetic tree construction. Sequences were aligned using Muscle, and neighbor-joining trees were constructed using the p-distance model with 500 bootstrap replicates. The accession number and protein sequence of other fish in the phylogenetic tree analysis are provided in Table S1.

#### 2.2.3. Detection of *kiss1* Expression in Different Tissues of *S. biddulphi*

Total RNA was extracted from the heart, liver, mid-kidney, muscle, gonad, eye, hypothalamus, pituitary gland, gill, fin, and swim bladder tissues of *S. biddulphi* (n = 3) using TRIzol reagent (Invitrogen, Waltham, MA, USA) according to the protocol of the US manufacturer. The amount of cDNA used in the qPCR system for different tissues was adjusted using β-actin as the internal reference gene. qPCR was performed according to the qPCR primers qkiss1-F and qkiss1-R, which were designed based on the *kiss1* gene sequence (Table 1). qPCR was performed using a 15 μL system: 2 × SG Green qPCR Mix 7.5 μL, 0.25 μL of each upstream and downstream primer (10 μM), 1 μL of cDNA, and 6 μL of ddH2O. The PCR parameters included initial denaturation at 95 °C for 10 min, denaturation at 95 °C for 20 s, and annealing and extension at 60 °C for 30 s for 40 cycles. After PCR was completed, the melting curve was analyzed to determine the amplification of individual products. Three parallel PCRs were performed for each tissue sample, and the results obtained were analyzed using the comparative CT method (2^−∆∆Ct^) to obtain the relative expression level of each tissue. Origin 2021 software was used to create graphs. 

#### 2.2.4. SNP Locus Screening of the *kiss1* Gene

Total RNA was extracted from each sample (n = 62) and reverse transcribed to cDNA as described above. Using this cDNA as a template, PCR amplification was performed using the *kiss1*-F and *kiss1*-R primers (Table 1). Primer design was performed on the NCBI website (http://www.ncbi.nlm.nih.gov/tools/primer-blast/index.cgi?LINK_LOC=BlastHome, accessed on 28 February 2022) based on the conserved sequence on either side of the site. The main parameters are as follows: the length of primer was generally 18~24 bp; Tm 55 °C~65 °C; (G+C) content 40%~70%; production size was less than 800 bp. The products were purified by gel cutting and ligation transformation, followed by direct sequencing. The specific steps were performed as described above. Sequencing results were analyzed with sequence alignment using DNAMAN (version v 10.0.2.128; Lynnon BioSoft, Vaudreuil, QC, Canada) to find SNP sites.

#### 2.2.5. Measurement of Reproductive Traits and Evaluation of its Association with SNP Loci

The gonadosomatic index (GSI) was calculated as follows: maturation coefficient = (gonadal weight/total weight) × 100%. The test data were expressed as the mean ± standard deviation (mean ± S.D.). The genotype frequency and allele frequency at each SNP locus of the *kiss1* gene were calculated using Microsoft Excel, and the Hardy–Weinberg equilibrium (HWE) test was performed using the *χ^2^* test. ANOVA was performed in SPSS 26.0 to calculate the associations between different genotypes of the *kiss1* gene and reproductive traits, and the results were expressed as the mean ± standard deviation. Duncan’s new complex polarization method (Duncan’s) was used to determine the significance of differences between the means of the groups, with significant differences indicated by *p* < 0.05.

## 3. Results

### 3.1. Sequence Analysis of the kiss1 Gene

**Figure 1:**
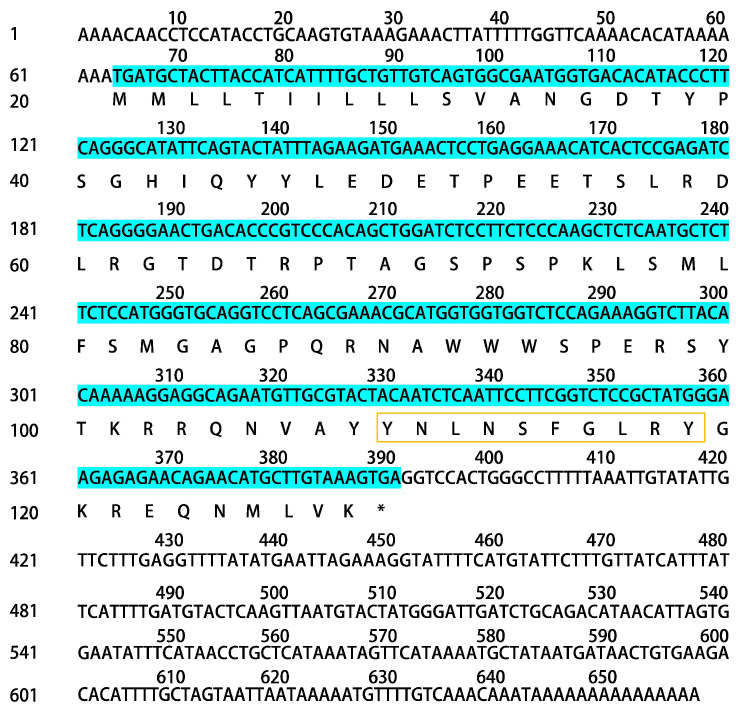
The *kiss1* nucleotide sequence and its amino acid sequence. Sequences in blue are ORF, * Stop codon, Sequences in the box are Kisspeptin1-10.

The *kiss1* gene was 658 bp long, with a full-length ORF of 327 bp, and encoded 108 amino acids. The 5′ UTR was 62 bp long, the 3′ UTR was 269 bp long, the start codon was ATG, and the stop codon was TGA. Underlined was Kisspeptin-10 (YNLNSFGLRY) (Figure 1).

There are 14 species that have greater than 80% amino acid sequence similarity with *S. biddulphi* (Table 2), which indicates that the protein encoded by the *kiss1* gene cloned from *S. biddulphi* was highly conserved with that of other species.

**Figure 2:**
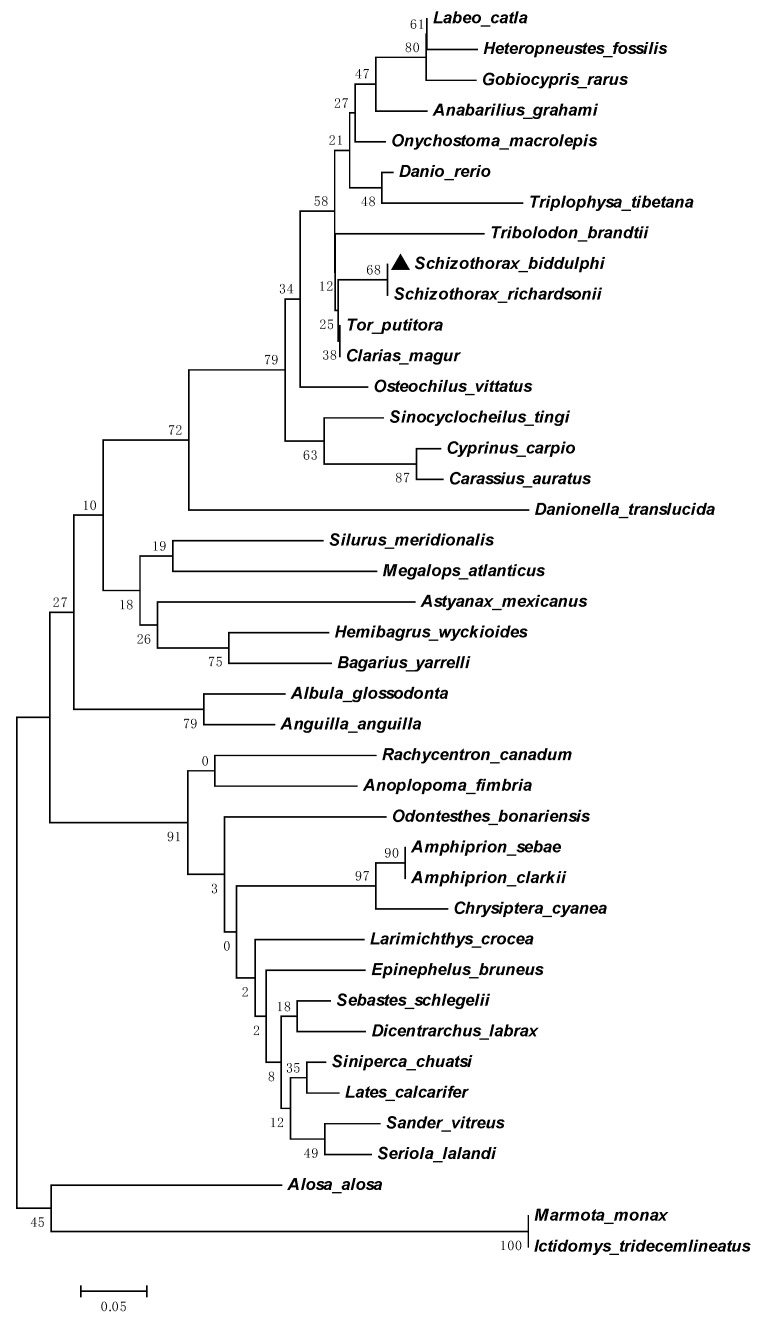
Phylogenetic tree analysis of the *kiss1* protein in different species. The number shown at each branch indicates the bootstrap value (%). GenBank accession numbers see Table 2.

The results of the evolutionary tree analysis of *kiss1* proteins (Figure 2) showed that the *kiss1* protein of *S. biddulphi* was most closely related to that of *S. richardsonii*. 

### 3.2. Characterization of kiss1-Encoded Proteins

**Figure 3:**
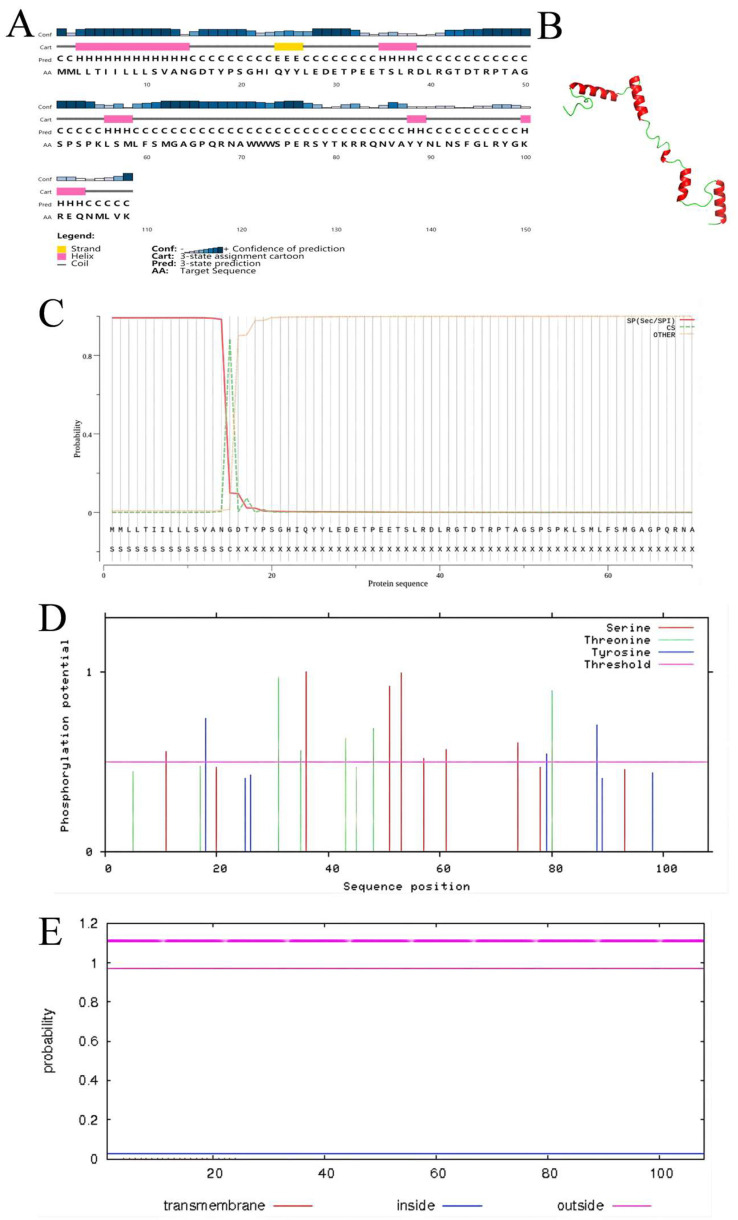
The characterization of *kiss1* protein (**A**), Secondary structure; (**B**), Tertiary structure; (**C**), Prediction of the signal peptide; (**D**), Phosphorylation sites prediction; (**E**), Transmembrane domain prediction).

The theoretical isoelectric point of the protein encoded by the *kiss1* gene is 9.34, and the molecular weight of the *kiss1* protein is 12.45 kDa. The 108 encoded amino acids comprise 19 unique amino acids, the most common of which is leucine (12.0%) and the least common of which is histidine (0.9%); there are 13 positively charged amino acids (Lys and Arg) (12.0%) and 10 negatively charged amino acids (Asp and Glu) (9.3%). The total number of protein atoms is 1733, and the instability index is 60.41. The protein is unstable. The secondary structure of the *kiss1* protein mainly contained two secondary components, the extended strand and random coil (Figure 3A), with the α-helix accounting for 23.15%, the extended strand accounting for 16.67%, and the random coil accounting for 60.19%. The similarity of the tertiary structure with this template was 45% (Figure 3B). The shear site of the signal peptide was located between amino acids 15 and 16 (Figure 3C). There were 10 serine phosphorylation sites located at sites 11, 20, 36, 51, 53, 57, 61, 74, 78, and 93; there were 8 threonine phosphorylation sites located at sites 5, 17, 31, 35, 43, 45, 48, and 80; and there were 7 tyrosine phosphorylation sites, located at sites 18, 25, 26, 79, 88, 89, and 98 (Figure 3D). The *kiss1* protein is not a transmembrane protein (Figure 3E), and its subcellular localization is mainly outside the cell; presumably, its function is performed mainly outside the cell.

### 3.3. Tissue Expression Profile of the kiss1 Gene

**Figure 4:**
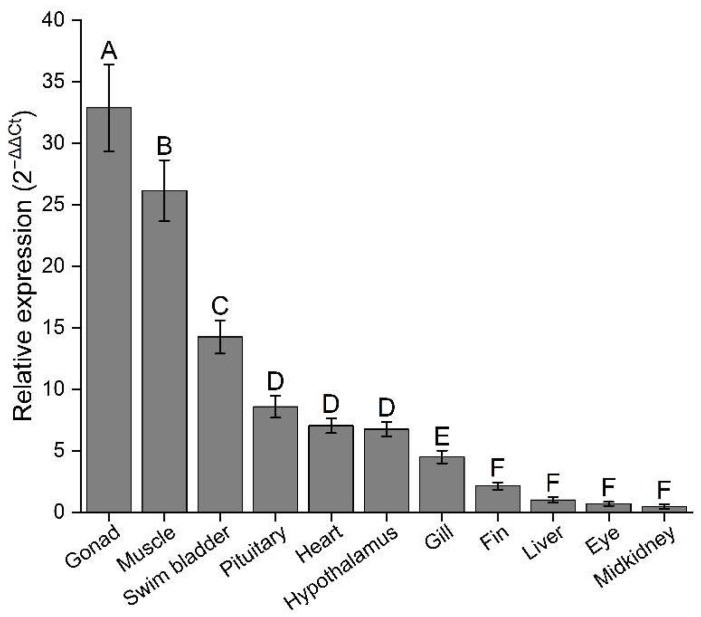
Distribution of the *kiss1* gene in different tissues of male *S. biddulphi.* Different letters indicate that the difference is significant, and the same letters indicate that the difference is nonsignificant (*p* > 0.05).

The expression of the *kiss1* gene in different tissues of male *S. biddulphi* was detected by fluorescence quantitative PCR, and the results showed that the *kiss1* gene was expressed in 11 tissues. *kiss1* gene expression was highest in the gonads of male *S*. *biddulphi*, followed by the muscle, and then was ordered from high to low as follows: swim bladder, pituitary gland, heart, hypothalamus, gill, fin, liver, eye, and mid-kidney (Figure 4). 

### 3.4. SNP Diversity Analysis of the kiss1 Gene

#### 3.4.1. Polymorphic SNP Loci of the *kiss1* Gene

Three SNP loci were detected in the exonic region of the *kiss1* gene, and their base changes and amino acid changes, mutation types, and genetic structure information are shown in Table 2. SNP c.3G>T and SNP c.198T>G both have GG and TT genotypes, of which TT is the dominant genotype, with two alleles, G and T, of which T is the dominant allele. SNP c.12T>C has two genotypes, CC and TT, where C is the dominant genotype. The mutation types at these three loci were all missense mutations, and all deviated from Hardy–Weinberg equilibrium (*p* < 0.05, Table 3).

#### 3.4.2. Association Analysis of SNP Loci of the kiss1 Gene with Reproductive Traits in *S. biddulphi* Males

The gonad weight and gonadosomatic index of individuals with the TT genotype were significantly higher than those of individuals with the GG genotype at SNP c.3G>T (*p* < 0.05). The gonad weight and gonadosomatic index of individuals with SNP c.12T>C and SNP c.198T>G were not significantly different (*p* > 0.05, Table 4). 

## 4. Discussion

The developmental process in animals is extremely complex and precise, and the central nervous system and the endocrine system work together to jointly regulate the developmental process, with the kisspeptin system playing a key role in reproductive development and energy metabolism in fish [28,37]. In this study, the 658-bp cDNA sequence of *kiss1* was cloned from *S. biddulphi* for the first time, which encoded a 108-amino acid protein. The C-terminal core decapeptide of this sequence is YNLNSFGLRY (Y-Y pattern), the shortest mature sequence that stimulates phosphatidylinositol conversion at the amino acid level [38], which is highly consistent with the C-terminal decapeptide sequences of *D. rerio* [39], *O. latipes* [40] and other carps, showing that the core sequence of the *kiss1* gene, kisspeptin-10, is highly conserved in carps. In contrast, in mammals and amphibians, the corresponding amino acid at position 3 of this core sequence is W [41,42]. In fish, it corresponds to L, immediately following the core decapeptide with GLR as the splitting site and amidation site of this sequence. This short peptide is equally highly conserved in mammals and fish [38,39]. *kiss1* and *kiss2* encode different functional proteins, and in the *kiss2* protein, the C-terminal core decapeptide YNLNSFGLRY of the *kiss1* protein corresponds to FNYNPFGLRF, where the same motif of -N-N-FGLR- is present [32,43]. The *kiss1* and *kiss2* genes are RY- and RF-amide-type compounds, respectively. Generally, only *kiss1* is present in mammals, and some scholars speculate that it may be due to the loss of the *kiss2* gene during evolution. Fish generally contain two *kiss* genes, *kiss1* and *kiss2*. *Anguilla anguilla* is the only teleost species having two kiss genes (*kiss1* and *kiss2*) and three kissr types (*kissr1*, *kissr2*, and *kissr3*) reported to date, indicating that the evolutionary process varies with species [13]. The secondary structure of the *kiss1* gene of *S. biddulphi* mainly includes an extended strand and irregular curl, while the irregular curl structure often dominates changes in enzyme activity and functional site composition [44], which is presumed to have an important role in the formation of specific functional sites, and its inclusion of two plus-tail signals (AATAAA) suggests selective shearing during transcription [45]. There were 14 fish species with an amino acid sequence similarity more significant than 80%, and the homology results showed that the *kiss1* amino acid sequence of *S. biddulphi* had the highest similarity of 98% with that of the congener *S. richardsonii*. In the phylogenetic tree, they were clustered onto one branch, indicating the closest genetic relationship.

Exploring the expression of a gene in different tissues helps analyze the mechanism of gene regulation, and the gene expression pattern indicates, to some extent, the functional diversity among tissues [23]. It has been confirmed that the kisspeptin system mediates the reproductive endocrine process in animals. In this study, the *kiss1* gene had the highest expression in the gonads, followed by the muscle, swim bladder, pituitary gland, heart, hypothalamus, and gill, and low expression in the fin, liver, eye, and mid-kidney, similar to the *kiss1* gene tissue expression results of *Acipenser schrenckii* [30]. In other teleosts, there were differences in the pattern of *kiss1* gene expression, with *D. rerio*’s *kiss1* being most expressed in the brain and pituitary gland and the intestine, pancreas, kidney, and adipose tissue [39]. *kiss1* of *S. japonicus* was expressed in the brain, gonads, gills, heart, and spleen, but *kiss1* expression was not present in the pituitary gland, and the pattern of expression varied by reproductive cycle [28]. In *T. putitora*, the expression of *kiss1* was inconsistent between ovarian development and spermatogenesis [46], and the expression of *kiss1* increased as the organism developed. These results indicate that the expression of *kiss1* varies among species, gender, and reproductive cycles. The high expression in the gonads of *S. biddulphi* is consistent with findings for *O. latipes* [40], *C. auratus* [47], and *S. japonicus* [28], confirming the involvement of *kiss1* in the reproductive function of fish. In some species, *Oryzias latipes*, zebrafish, *Dicentrarchus labrax,* and *Scomber japonicus*, it has been demonstrated that *kiss1* is regularly expressed at different developmental stages in the male brain [23,40,48,49]. In the brain of *Scomber japonicus* males, *kiss1* mRNA expression level gradually decreased from the immature stage to the spermiation stage, followed by a significant decline during the post-spawning stage [23]. The *kiss1* gene in the brain of *S. biddulphi* may follow a similar expression pattern during the gonadal development stage. In the present study, the *kiss1* gene expression levels in the pituitary and hypothalamus were not very high, probably because gonads were developed to stage IV or V. They may not be at the peak of expression in the temporal expression pattern.

In addition to the gonads, *kiss1* is also highly expressed in muscle tissues. It has been confirmed that muscle tissue is one of the main regions for energy accumulation and metabolism, and the *kiss1* gene has an important role in maintaining energy balance [24]. Kisspeptin can act as an anorexigenic factor, but its effect on energy balance is not mediated by food intake but by regulating energy digestion, which is related to the number of kisspeptin^ARC^ neurons and can have an effect on body weight changes in mice [21,50]. The results of a large number of metabolic stress models show that reproductive regulation is inhibited by the metabolic stress response, and leptin and NPY factors, which are closely related to energy metabolism, can inhibit the expression of the *kiss* gene, which in turn affects the reproduction of the organism [51]. However, the specific controlling mechanisms still need to be investigated. The pituitary gland, as an indispensable component of the HPG axis, mutually regulates its function with the hypothalamus and gonads. The hypothalamus releases gonadotropin-releasing hormone (GnRH) that binds to receptors that promote the release of luteinizing hormone (LH) and follicle-stimulating hormone (FSH) from the pituitary gland, forming a neuroendocrine network that ultimately affects the reproductive function of fish and this series of cascade reactions cannot be separated from either organ. The expression of *kiss1* in the pituitary gland and hypothalamus is also consistent with their respective functions [52]. Similar results were obtained for *O. latipes* [40], *C. auratus* [47], *L. rohita* [53], *D. labrax* [21], etc. Interestingly, *kiss1* gene expression was also detected in the fins of fish, which play an important role in maintaining balance and assisting forward movement during swimming, suggesting that *kiss1* has some function other than regulating reproduction.

Reproductive traits are important indicators for assessing the reproductive potential of individuals. The important role of *kiss1* in animal reproduction can make it a candidate gene for fertility, and by identifying its SNP loci and analyzing the association between genotype and phenotype, effective molecular markers for the genetic improvement of animal reproductive traits can be obtained [54]. The SNP g.1311578G>T locus found in *Ovis aries* plays a key role in lambing and can be screened for the TT dominant genotype to improve reproductive and production potential [55]. Maitra et al. (2014) found three SNP loci (G296C, G2510A, and C2540T) related to the litter size of Indian *C. hircus* in intron 1 of the *kiss1* gene [56]. An et al. (2013) detected two SNP loci (G2124T>A and G2270C>T) significantly associated with the lambing number of *C. hircus* in intron 1 of the *kiss1* gene in three different sheep populations [57]. Similar mutations at the G227C and T486A loci of the *kiss1* gene were reported to be associated with lambing numbers in Northern Guizhou *C. hircus* [58].

In this study, for the first time, SNP loci in exons of the *kiss1* gene were correlated with reproductive traits in male *S. biddulphi*, and the results showed that the gonad weight and maturation coefficients of individual *S. biddulphi* males with the TT genotype were significantly higher than those of individuals with the GG genotype at SNP c.3G>T. Mutation of single nucleotides at the SNP c.3G>T locus resulted in amino acid types that significantly affected gonad mass and maturation coefficients of *S. biddulphi* males. The exonic region belongs to the coding region of the gene, and this single base substitution leads to an amino acid change; this nonsynonymous mutation may affect the stability of the protein and function of the gene, which may be responsible for the differences in reproductive traits among individuals; therefore, it is hypothesized that the dominant allele T improves the reproductive ability of *S. biddulphi* to some extent. The results also indicated that the pure TT genotype at the SNP c.3G>T locus in *S. biddulphi* yielded better reproductive traits and could be used to select parents for breeding progeny with obvious reproductive advantages. Since reproductive traits are influenced not only by genes but also by the environment, it is not clear whether the *kiss1* gene is the main-effect gene affecting *S. biddulphi* or whether there is perhaps a linkage with other main-effect genes controlling reproductive traits, and the mechanism needs to be further explored. 

Although the size of the studied population was relatively small, the present results have important and practical implications for the recovery and continuation of resources of *S. biddulphi* and the breeding of superior breeds, which may lead to significant improvement in reproductive characteristics. However, differences in gonad weight and maturation coefficients among genotypes need to be confirmed based on a larger amount of additional data to confirm their significant effects. The next step could be the identification and screening of associated SNP loci for highly reproductive females, which could be applied in actual reproduction practices in combination with the present results.

## 5. Conclusions

In conclusion, the *kiss1* gene of *S. biddulphi* is homologous to that in other fishes, and the widespread expression of *kiss1* in different tissues implies that it may be involved in functions other than reproduction. However, in-depth research is still needed regarding its expression pattern and involvement in other functions during gonad development. In this study, we searched for mutated loci of *kiss1* in *S. biddulphi* and found that the SNP c.3G>T locus was significantly associated with both gonad mass and the maturation coefficient in *S. biddulphi*, and individuals with the genotype TT had significantly larger reproductive traits than those with other genotypes. The identified SNPs could be used as markers for higher fertility and, thus, to improve the reproductive performance of this population.

## Figures and Tables

**Table 1:** Primer sequences for amplification of the *kiss1* gene.

Primer ID	Primer Sequence (5′-3′)	Use
mkiss1-F	GAATGGTGACACATACCCTTCAG	Coding region
mkiss1-R	GACCTTTCTGGAGACCACC	Coding region
rkiss1-R1	GGACGGGTGTCAGTTCCCCTGAGATC	5′ RACE
rkiss1-R2	CGGAGTGATGTTTCCTCAGGAGTTTC	5′ RACE
rkiss1-F1	GACACATACCCTTCAGGGCATATTCAGTAC	3′ RACE
rkiss1-F2	GATGAAACTCCTGAGGAAACATCACTCCG	3′ RACE
qkiss1-F	ATTTTCATGTATTCTTTGTT	qPCR
qkiss1-R	GTCTGTATTGTAATCACCTT	qPCR
β-actin-F	AATCCCAAAGCCAACAGA	qPCR (Internal control)
β-actin-R	CGACCAGAAGCGTACAGAG	qPCR (Internal control)
kiss1-F	ACAACCTCCATACCTGCAAGTG	SNP Screening
kiss1-R	ATGAGCAGGTTATGAAATATTCCA	SNP Screening

**Table 2:** Amino acid similarity levels of the kiss1 protein of *S. biddulphi* with those from other species.

Species	Per. Ident	Accession Number
*Labeo catla*	88.79%	AIZ66894.1
*Heteropneustes fossilis*	84.21%	QPD01600.1
*Gobiocypris rarus*	82.86%	AHH83757.1
*Anabarilius grahami*	88.68%	ROL46005.1
*Onychostoma macrolepis*	92.59%	KAF4107520.1
*Danio rerio*	82.41%	ACT10282.1
*Triplophysa tibetana*	64.29%	KAA0707738.1
*Tribolodon brandtii*	83.78%	ASU91841.1
*Schizothorax richardsonii*	98.04%	AIL56343.1
*Tor putitora*	94.74%	AIZ03572.1
*Clarias magur*	93.22%	AWK21963.1
*Osteochilus vittatus*	85.51%	QKG01959.1
*Sinocyclocheilus tingi*	86.11%	APT42866.1
*Cyprinus carpio*	85.19%	KTG38456.1
*Carassius auratus*	82.86%	ACK77790.1
*Danionella translucida*	47.17%	TRZ01864.1
*Silurus meridionalis*	43.86%	XP_046730475.1
*Megalops atlanticus*	40.59%	KAG7477746.1
*Astyanax mexicanus*	41.75%	XP_049327815.1
*Hemibagrus wyckioides*	43.43%	KAG7319116.1
*Bagarius yarrelli*	38.03%	TST85788.1
*Albula glossodonta*	40.91%	KAG9352792.1
*Anguilla anguilla*	40.70%	XP_035245162.1
*Rachycentron canadum*	31.37%	ANJ46819.1
*Anoplopoma fimbria*	33.98%	AKN78944.1
*Odontesthes bonariensis*	43.66%	AHA46378.1
*Amphiprion sebae*	30.10%	AJP70562.1
*Amphiprion clarkii*	30.10%	QID05230.1
*Chrysiptera cyanea*	30.10%	BAO21623.1
*Larimichthys crocea*	39.36%	TMS19587.1
*Epinephelus bruneus*	34.29%	ADF59544.1
*Sebastes schlegelii*	35.78%	AIZ68243.1
*Dicentrarchus labrax*	31.43%	ACM07422.1
*Siniperca chuatsi*	34.62%	QYY49470.1
*Lates calcarifer*	31.37%	XP_050930503.1
*Sander vitreus*	33.98%	AFV25604.1
*Seriola lalandi*	28.16%	AEF32393.1
*Alosa alosa*	36.00%	KAG5274662.1
*Ictidomys tridecemlineatus*	38.16%	XP_005330451.2
*Marmota monax*	38.16%	XP_046319241.1

**Table 3:** SNP locus information of the *kiss1* gene.

SNPs	Amino Acid Change	Mutation Type	Genotype Frequency	Gene Frequency	*p* Value
SNP c.3G>T	M→Y	missense mutation	GG (0.20) TT (0.80)	G (0.20) T (0.80)	<0.01
SNP c.12T>C	G→Q	missense mutation	CC (0.80) TT (0.20)	C (0.80) T (0.20)	<0.01
SNP c.198T>G	P→G	missense mutation	GG (0.12) TT (0.87)	G (0.125) T (0.875)	<0.01

**Table 4:** Association analysis between *kiss1* polymorphism and reproductive traits.

SNPs	Genotype	Gonad Weight	Gonadosomatic Index
SNP c.3G>T	TT	1.39 ± 0.16 ^a^	1.75 ± 0.01 ^a^
	GG	0.73 ± 0.27 ^b^	1.70 ± 0.02 ^b^
SNP c.12T>C	CC	1.27 ± 0.14 ^a^	1.74 ± 0.01 ^a^
	TT	0.73 ± 0.26 ^a^	1.70 ± 0.02 ^a^
SNP c.198T>G	TT	1.22 ± 0.14 ^a^	1.73 ± 0.01 ^a^
	GG	0.72 ± 0.32 ^a^	1.69 ± 0.03 ^a^

In the same SNPs with same small letter superscripts mean no significant differences (*p* > 0.05); different small letter superscripts mean significant differences (*p* < 0.05).

## Data Availability

The original contributions presented in the study are included in the article. Further inquiries can be directed to the corresponding authors.

## Supplementary Materials

The following supporting information can be downloaded at: https://www.mdpi.com/article/10.3390/genes14040862/s1, Table S1: The accession number and protein sequence of other fish in the phylogenetic tree analysis.
